# Magnesium corrosion particles do not interfere with the immune function of primary human and murine macrophages

**DOI:** 10.1007/s40204-014-0032-9

**Published:** 2014-12-06

**Authors:** Isabelle Roth, Stephan Schumacher, Tina Basler, Kathrin Baumert, Jan-Marten Seitz, Florian Evertz, Peter Paul Müller, Wolfgang Bäumer, Manfred Kietzmann

**Affiliations:** 1 Institute of Pharmacology, Toxicology and Pharmacy, University of Veterinary Medicine Hannover, Foundation, Bünteweg 17, 30559 Hannover, Germany; 2 Institute of Microbiology, University of Veterinary Medicine Hannover, Foundation, Bünteweg 17, 30559 Hannover, Germany; 3 Division of Immunodermatology and Allergy Research, Clinic for Dermatology, Allergy and Venereology, Hannover Medical School, Carl-Neuberg-Str. 1, 30625 Hannover, Germany; 4 Institute of Materials Science, Leibniz Universität Hannover, An der Universität 2, Garbsen, 30823 Hannover, Germany; 5 Institute of Multiphase Processes, Leibniz Universität Hannover, Callinstr. 36, 30167 Hannover, Germany; 6 Helmholtz Centre for Infection Research, Inhoffenstraße 7, 38124 Brunswick, Germany; 7 MBS Department, NCSU College of Veterinary Medicine, Research Building, Office 452, Lab 218, 1060 William Moore Drive, Raleigh, NC 27607 USA; 8 Present Address: Elanco Animal Health, Lilly Deutschland GmbH, Werner-Reimers-Str. 2-4, 61352 Bad Homburg, Germany

**Keywords:** Degradation, Immunotoxic, Infection, Inflammation, In vitro, Phagocytes

## Abstract

Magnesium is currently under investigation as a prospective biodegradable implant material. Biodegradation of magnesium causes a release of magnesium, hydroxide ions and hydrogen gas but it can also lead to the formation of particulate debris. Implant-derived particles may have immunotoxic effects. To investigate the influence of magnesium-derived particles on the immune functions of primary macrophages, up to 500 μg/ml magnesium or magnesium corrosion particles were added to the cell culture medium. No major effects were observed on cell viability and on the release of the proinflammatory cytokine tumor necrosis factor (TNF)α. In addition, the ability of macrophages to stimulate proliferation of allogenic lymphocytes in a mixed leukocyte reaction remained unaffected. When macrophages were incubated with magnesium particles and then infected with the apathogenic *Mycobacterium smegmatis*, infection-induced TNFα secretion from murine macrophages was inhibited but not from human macrophages. However, the bactericidal activity of either cell type was not influenced. In conclusion, magnesium-related particles did not restrict the immune function of macrophages, suggesting that magnesium implants and corrosion particles derived thereof are highly biocompatible and have a low inflammatory potential.

**Electronic supplementary material:**

The online version of this article (doi:10.1007/s40204-014-0032-9) contains supplementary material, which is available to authorized users.

## Introduction

The development of magnesium-based implants has become an intense focus of biomedical research in recent years (Gu et al. 2009; Moravej and Mantovani 2011; Purnama et al. 2010; Waizy et al. 2013; Witte et al. 2008). These implants are biodegradable and offer a number of unique advantages for temporary applications such as bone-healing supports. After the healing process is completed the implant would disintegrate and the surgery for implant removal would no longer be required, with major benefits for the patient as well as by reducing the burden on the healthcare system, thus saving both risks and costs (Kraus et al. 2012; Staiger et al. 2006). Moreover, permanent implant materials and, in particular, implant wear particles can elicit an inflammatory foreign body response (Bondarenko et al. 2011; Hallab and Jacobs 2009; Witte et al. 2007). Such long-term side effects could be avoided with self-degradable implants. Magnesium and its alloys are exceptional as potential implant materials since the mechanical properties are more similar to cortical bone than polymers or permanent alloys, which could minimize stress-shielding effects (Staiger et al. 2006). And finally, self-degrading implants could avoid mechanical stress if permanent implants were not be removed from pediatric patients where the tissues are still growing (Hermawan et al. 2010; Kraus et al. 2012). These properties explain why magnesium-based biomaterials are primarily investigated for temporary applications such as orthopedic and cardiovascular implants, e.g., osteosynthetic screws and plates, intramedullary nails or vessel stents (Hermawan et al. 2010; Waizy et al. 2013).

However, biodegradability may also pose a problem since magnesium corrosion may result in particulate debris and implant-derived corrosion particles may induce pain and inflammation. Most studies reported appropriate immunological response to magnesium implants (Badar et al. 2013; Bondarenko et al. 2011; Feser et al. 2011; Kraus et al. 2012; Willbold et al. 2011; Witte et al. 2007) but excess degradation rates have been associated with increased immune cell infiltration (Dziuba et al. 2013; Erdmann et al. 2010). In a recent study the bone tissue response was evaluated after complete degradation of a ZEK100 magnesium alloy implant and the authors reported severe pathological alterations with elevated numbers of inflammatory cells, such as osteoclasts, macrophages and giant cells (Dziuba et al. 2013). Both ion release and wear debris from permanent implants can activate macrophages and osteoclasts to induce inflammation and osteolysis and eventually lead to implant failure (Hallab and Jacobs 2009). Co–Cr–Mo alloy particles have been shown to increase the release of inflammatory cytokines such as TNFα from monocytes and macrophages (Caicedo et al. 2009, 2010) and induce apoptosis of cultured osteocytes (Kanaji et al. 2009). Likewise, titanium particles were shown to have negative effects on osteoblasts and macrophages (Fritz et al. 2002; Lee et al. 2012; Mao et al. 2012). Considering that magnesium-containing microparticles have been reported to be released from corroding implants in vivo (Badar et al. 2013) and the fact that they have also been observed in phagocytes (Bondarenko et al. 2011; Witte et al. 2007), it is mandatory to assess the potential of magnesium-derived particles for immunological effects. Little is known about cytotoxic and genotoxic effects of magnesium particles (Di Virgilio et al. 2011; Grillo et al. 2011). Magnesium ions and degradation extracts do not appear to interfere significantly with antigen-presenting cells (Feser et al. 2011) but so far the immunotoxic potential of particulate corrosion products has not been assessed. The aim of this study was to investigate the effects of magnesium particles as well as magnesium corrosion particles on primary cells of the innate immune system and elucidate whether their immunocompetence might be affected.

## Materials and methods

### Primary murine macrophages

Husbandry and handling of mice used for the generation of cells was done according to German animal welfare regulations. Bone marrow-derived macrophages were generated from the bone marrow of 8–20-week-old female BALB/c mice using a modified version of the protocol of Lutz et al. (1999). Briefly, the femur was explanted, both ends were cut and the bone marrow was flushed with 5 ml sterile phosphate-buffered saline (PBS; 137 mM NaCl, 2.7 mM KCl, 8 mM Na_2_HPO_4_, 1.7 mM KH_2_PO_4_, pH 7.4; salts from Merck KGgA, Darmstadt, Germany). The cells were then pelleted via centrifugation and resuspended in culture medium consisting of RPMI 1640 medium (Biochrom AG, Berlin, Germany) supplemented with 10 % FBS, 100 IU/ml penicillin, 100 µg/ml streptomycin (all PAA Laboratories, Pasching, Austria) and 50 µM 2-mercaptoethanol (Sigma–Aldrich GmbH, Steinheim, Germany). Cells were then seeded in a 10-cm dish (Cell+, Sarstedt AG, Nümbrecht, Germany) at a density of approx. 40,000/cm^2^ and stimulated with 20 ng/ml murine granulocyte macrophage colony-stimulating factor (GM-CSF, R&D Systems, Wiesbaden, Germany) to induce differentiation into dendritic cells and macrophages. Culture was continued for 9 days and fresh culture medium with GM-CSF was added on days 3, 6 and 8. On day 9, the supernatant containing the dendritic cells was removed and RPMI containing FBS and antibiotics was added. The adherent macrophages were scraped off the dishes on day 14, reseeded in a 12-well plate (Greiner Bio-One GmbH, Frickenhausen, Germany) at 200,000 cells/well in 1 ml medium and incubated for at least 4 h to assure attachment before experiments were started.

### Primary human macrophages

Peripheral blood mononuclear cells (PBMC) were isolated from the buffy coats of peripheral blood from anonymous healthy donors (kindly provided by the Hannover Medical School) according to Kasraie et al. (2010) with some modifications. Briefly, PBMC were obtained via density-gradient centrifugation using Pancoll human (PAN-Biotech, Aidenbach, Germany) and seeded in a 10-cm dish at approximately 160,000 cells/cm^2^ in Iscove Basal Medium (Biochrom) supplemented with 4 % human AB serum (PAN-Biotech), 1 % non-essential amino acids (Biochrom), 2 mM l-glutamine (PAA), 100 IU/ml penicillin, 100 μg/ml streptomycin and 50 μg/ml gentamicin (PAA). Cells were incubated at 37 °C and 5 % CO_2_ for 1 h before washing five times with sterile PBS. The remaining adherent cells were mainly monocytes which were then incubated for 7 days in RPMI medium (PAA) containing 5 % FCS, 12 mM HEPES (Life Technologies, Darmstadt, Germany), 2 mM l-glutamine, 100 IU/ml penicillin, 100 μg/ml streptomycin and 10 ng/ml granulocyte macrophage colony-stimulating factor (Leukine, Bayer HealthCare Pharmaceuticals, Berlin, Germany) to induce differentiation into macrophages.

After this period, cells were collected via scraping and reseeded in a 12-well plate at 200,000 cells/well in 1 ml medium and incubated for at least 4 h to assure attachment. Non-adherent cells were removed with the medium and experiments were started.

### Treatment

Murine macrophages were treated with 20, 50, 100 or 500 µg/ml pure magnesium (Mg) particles (Omikron, Neckarwestheim, Germany) or magnesium corrosion particles (MCP) derived from pure Mg granulate (Riedel–de Haen, Seelze, Germany). To mimic magnesium degradation under physiological conditions 1 g Mg granulate was filled in a 15-ml Falcon tube and 5 ml PBS was added. The corrosion was allowed to proceed at ambient temperatures for 3 days. Then the supernatant was discarded and the remaining metallic magnesium together with the insoluble corrosion products was allowed to dry at ambient conditions. EDX analysis (Hitachi S-3400 scanning electron microscope, 15 keV, viewing distance about 10 mm) of the resulting powder detected Mg and O exclusively. Human macrophages were treated with 5, 10, 50, 100 or 500 µg/ml Mg, MCP or cobalt–chromium–molybdenum (CCM) particles. In some experiments cells were stimulated with 1 μg/ml lipopolysaccharide (LPS) O127:B8 (Sigma–Aldrich) to confirm TNFα-releasing capacity (results not shown).

After 24 h of incubation culture supernatants were collected, centrifuged at 3,000*g* for 5 min at 4 °C and the resulting supernatant was stored at −80 °C until further analysis.

### Scanning electron microscopy (SEM)

The structure of the different particles was determined using a JXA-8900R electron probe microanalyser (EPMA; JEOL GmbH, Eching, Germany). Here, the Rutherford backscattering spectrometry (RBS) mode was chosen to obtain best results. To determine a single grain’s dimension, self-adhesive carbon pads (Lighttabs; Plano GmbH, Wetzlar, Germany) were strewn with the particles resulting in a singular layer on the pad’s surface. Using a magnification of 200× or 2500×, respectively, single grains became clearly visible. Dimensions of the grains were measured using the microanalyser’s integrated measurement tool.

### Magnesium concentration, metabolic activity and cytokine analysis

Supernatant magnesium concentration was measured colorimetrically using a commercial assay kit (Nanocolor Härte 20, Macherey–Nagel, Düren, Germany) as previously described (Schumacher et al. 2011). To determine cell viability after particle treatment particle medium was removed and fresh medium supplemented with 20 % CellTiter96 AQueous One Solution (Promega, Mannheim, Germany) was added to the culture wells and incubated for 1 h at 37 °C at 5 % CO_2_. During this period metabolic activity of living cells led to a color change which was then quantified by measuring optical density at 490 nm using a microplate reader (MRX; Dynatech, Denkendorf). Murine tumor necrosis factor alpha (TNFα) or human TNFα, concentrations were determined using commercial assay kits (DuoSet ELISAs; R&D Systems, Wiesbaden, Germany) according to the manufacturer’s instructions. In cases where the calculated concentrations were below the limit of quantification of the assay kits, a value of (0.5 x lowest standard concentration of 31.25 pg/ml) was used for the statistical calculations. This applied to 26 out of 60 samples measured with the murine ELISA.

### Mixed leukocyte reaction

T cells were isolated from the spleens of female NMRI mice as previously described (Baumer et al. 2010). Briefly, spleens were flushed with sterile PBS, the cell suspension was centrifuged and the pellet resuspended in an erythrocyte lysis buffer (155 mM NH_4_Cl, 0.1 mM Na_2_EDTA, 10 mM NaHCO_3_, pH 7.3) and incubated for 5 min. After two washing steps cells were seeded in RPMI (PAA) containing 10 % FBS, 100 IU/ml penicillin and 100 μg/ml streptomycin in a 10-cm dish and incubated for 2–3 h to enrich T cells in the supernatant. Non-adherent cells were then collected and stained with 0.5 µM carboxyfluorescein succinimidyl ester (CFSE; Invitrogen, Karlsruhe, Germany) for 10 min followed by three washings. Murine macrophages were isolated as described above and incubated for 24 h with Mg, MCP or CCM particles at a concentration of 100 µg/ml. 10^4^ macrophages were then incubated with 10^5^ CFSE-stained lymphocytes in a U-bottom 96-well plate (Greiner) for 4–5 days before T cell proliferation was analyzed by flow cytometry (Coulter Epics XL and CXP Analysis software, Beckman Coulter, Miami, USA).

### Evaluation of immunocompetence

Immune function of murine and human macrophages was tested using a modified version of the protocol according to Kuehnel et al. (2001). After 24-h incubation of the cells with different particle concentrations, supernatants were removed and cells were infected with apathogenic *Mycobacterium smegmatis* [*M. smegmatis*; strain mc^2^155 (ATCC 19420)] which was cultured and prepared for infection as described previously (Kuehnel et al. 2001). Macrophages were incubated with a mycobacterial suspension of an OD_660 nm_ of 0.2 in antibiotic-free medium representing MOI of ∼10:1 for 2 h to allow intracellular penetration. After that, non-ingested mycobacteria were removed by several washing steps and remaining extracellular mycobacteria were killed by addition of 100 µg/ml gentamicin (Carl-Roth, Karlsruhe, Germany) for 1 h before cells were incubated with the respective treatment particle suspensions for further 20 h. Then, culture supernatants were again collected for cytokine measurements and macrophages were scraped and lysed in 1 % sodium dodecyl sulfate (SDS, Sigma–Aldrich) in PBS using a 23G needle (B. Braun, Melsungen, Germany) to release phagocytosed mycobacteria. Lysates were seeded on lysogeny broth (LB) agar plates (10 g/l tryptone, 5 g/l yeast extract, 10 g/l NaCl, 10 g/l agar–agar; Carl-Roth, Karlsruhe, Germany) and incubated for 6 days at 37 °C and 5 % CO_2_ before colony-forming units were counted. The initial mycobacterial suspension for infection was diluted and plated equally as a positive control.

### Statistical analysis

The treatment groups were checked for statistically significant differences using one-way ANOVA followed by Dunnett’s multiple comparison post hoc test. Statistical analysis was done using Prism software 5.01 (GraphPad Software, La Jolla, USA) and results of *p* < 0.05 were considered statistically significant.

## Results

### Biocompatibility

**Fig. 1:**
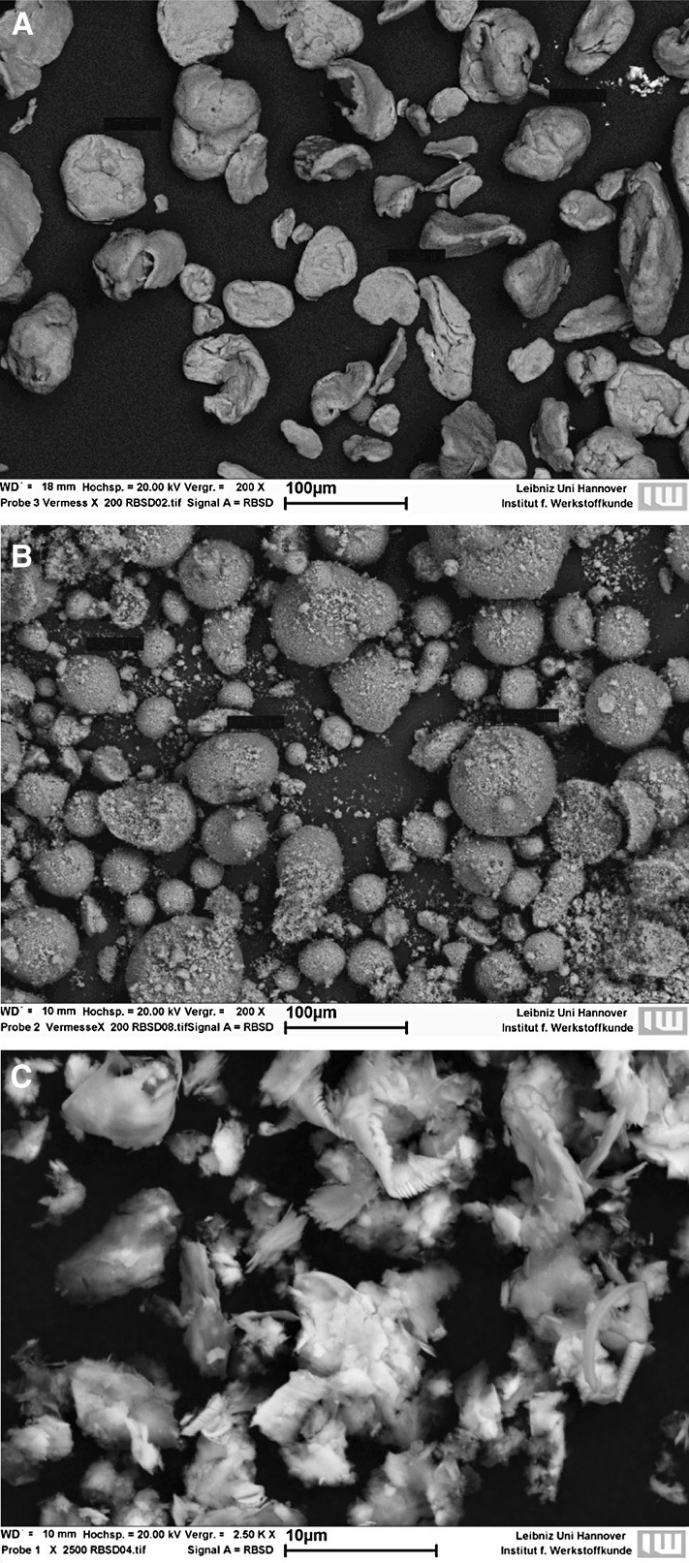
SEM images of particles. Scanning electron microscopy images of Mg particles (**a**), magnesium corrosion particles (**b**) and CCM particles (**c**) at 200× (**a**, **b**) or 2500× (**c**) magnification, respectively

SEM analysis revealed that Mg and magnesium corrosion particles were more or less round with an approximate size between 20 and 100 μm, although we also observed some MCP that were well below 10 μm. In contrast, the majority of CCM particles were smaller than 10 μm and their surface was much rougher and sharp edged (Fig. 1).

**Fig. 2:**
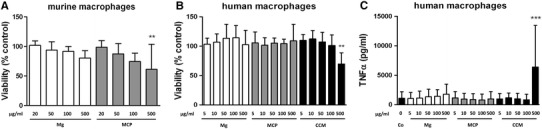
Biocompatibility of particles. Murine or human macrophages were incubated for 24 h with media containing indicated amount of magnesium particles (Mg), magnesium corrosion particles (MCP) or cobalt–chromium–molybdenum particles (CCM). Untreated cells served as a control (Co). **a** Metabolic activity, mean ± SD of *n* = 3–6. **b** Metabolic activity, mean ± SD of *n* = 8. **c** TNFα concentration, mean ± SD of *n* = 7. ANOVA, ** *p* < 0.01, *** *p* < 0.001 compared to control cells

To test for a cytotoxic effect of the particles we determined metabolic activity as a parameter for cell viability. After 24 h of incubation with different particle concentrations, metabolic activity of both murine and human macrophages remained largely unchanged with the exception of the 500 μg/ml doses of MCP in murine and CCM particles in human cells, which led to a 39 or 30 % reduction in viability, respectively (Fig. 2a, b). This result was contrary to the degree of degradation as higher amounts of magnesium ions were released from Mg versus magnesium corrosion particles (Table 1) and the pH was also slightly higher (8.36 vs. 8.19 for the 500 μg/ml doses).

**Table 1:** Magnesium concentration of treatment supernatants

Treatment (μg/ml)	Mg				MCP			
	20	50	100	500	20	50	100	500
Magnesium conc. (mM)	0.5 ± 0.2	1.2 ± 0.5	2.6 ± 0.6	10.8 ± 2.8	0.4 ± 0.3	0.6 ± 0.6	1.7 ± 1.9	3.6 ± 0.5

Magnesium concentration of supernatants after treatment with particle media containing indicated amount of magnesium-based particles, Mean ± SD of *n* = 3

We next determined the concentration of TNFα in the culture supernatants to assess whether the particles would elicit an inflammatory response. Unstimulated cytokine secretion from murine macrophages was below the limit of detection for all treatments. In contrast, human macrophages produced measurable amounts of TNFα but only the highest concentration of CCM particles (500 μg/ml) caused a significant increase (Fig. 2c).

### Immune function

**Fig. 3:**
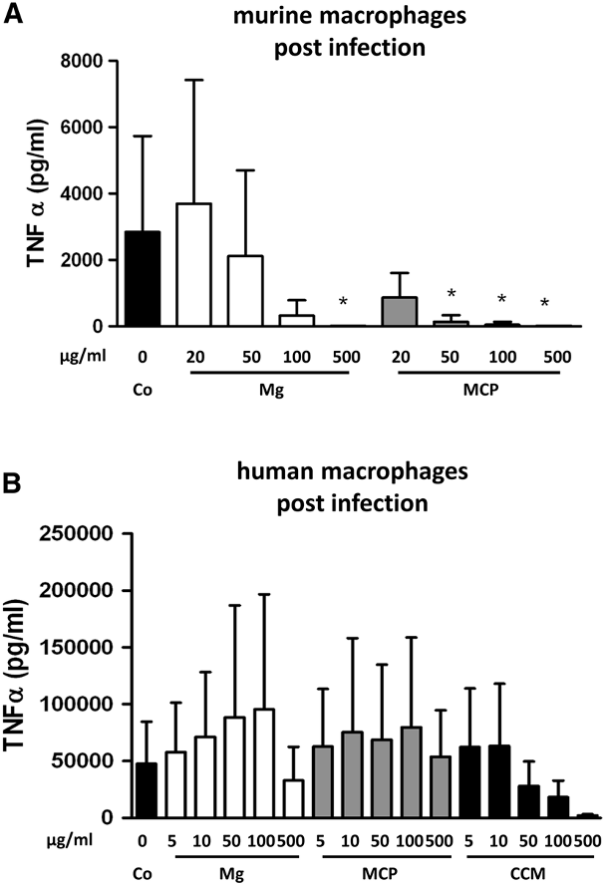
TNFα release from infected macrophages. Murine or human macrophages were incubated for 24 h with media containing either no particles (Co) or indicated amount of magnesium particles (Mg), magnesium corrosion particles (MCP) or cobalt–chromium–molybdenum particles (CCM) before infection with *Mycobacterium smegmatis*. Particle incubation was then continued for further 20 h. **a** Mean ± SD of *n* = 6–8. **b** Mean ± SD of *n* = 5. ANOVA, *p < 0.05 compared to untreated control cells. Changes in *panel b* were not statistically significant

Macrophages have very important functions as cells of the innate immune system. Infection of macrophages with the intracellular mycobacterium *M. smegmatis* leads to an immune response with increased production of TNFα (Roach and Schorey 2002), so we tested whether particle incubation would diminish this effect. Indeed, Mg and magnesium corrosion particles dose dependently reduced cytokine release from murine macrophages, with MCP being the more potent agent (Fig. 3a). Notably, viability of murine macrophages was decreased by about 50 % after *M. smegmatis* infection of cells that had been treated with MCP or 100–500 μg/ml Mg particles, indicating that the combined effect of these two stressors overwhelmed resistance of the cells and that MCP had a stronger negative impact (see Additional file 1).

Interestingly, the result was completely different when human cells were infected since magnesium-based particles did not lead to significant inhibition (Fig. 3b). The higher concentrations of CCM particles, however, resulted in decreased cytokine production, although this finding was not statistically significant due to the noise in the data.

**Fig. 4:**
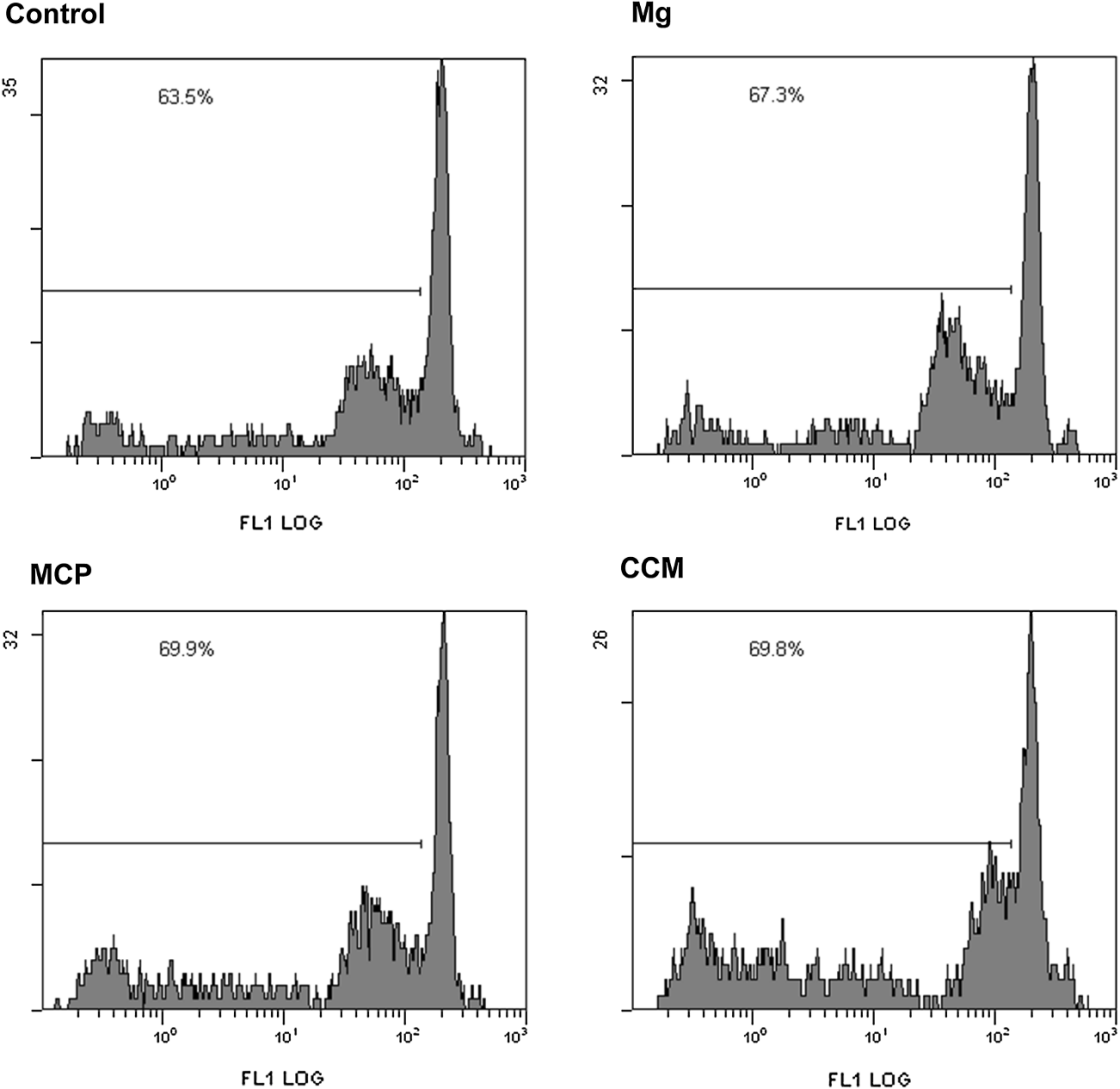
Mixed leukocyte reaction. Murine macrophages were treated for 24 h with media containing 100 μg/ml of indicated particles or media without particles before having been incubated with CFSE-stained lymphocytes. Representative histograms of three independent experiments showing percentage of proliferated cells

Beside their innate immune function macrophages can also act as antigen-presenting cells and thus induce the proliferation of allogenic T cells in a mixed leukocyte reaction. We investigated whether abraded magnesium degradation products might interfere with this process. Neither the two kinds of magnesium particles nor the CCM particles led to a significant change of T cell proliferation (Fig. 4; Table 2).

**Table 2:** Mixed leukocyte reaction

Treatment	Control (untreated)	Mg	MCP	CCM
Proliferated cells (%)	62.7 ± 0.9	65.2 ± 2.0	66.6 ± 5.7	66.0 ± 4.2

Proliferated lymphocytes after coincubation with particle-treated macrophages, Mean ± SD of *n* = 3

**Fig. 5:**
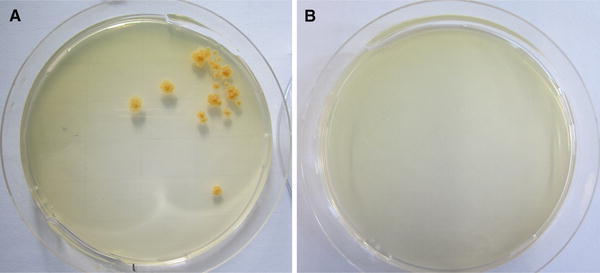
Absence of mycobacterial growth. Human macrophages were incubated for 24 h with media containing indicated amount of particles before infection with *Mycobacterium smegmatis*. Particle incubation was then continued for further 20 h before cells were lysed, seeded on Lysogeny broth agar plates and incubated for 6 days. *Panel a* shows growth of *Mycobacterium smegmatis*. The plate in *panel b* is representative of at least five independent experiments using human or murine macrophages, respectively. No colony-forming units were observed in any of the experiments after any particle treatment

To fight a mycobacterial infection macrophages need to be able to kill invading mycobacteria. Macrophages pretreated with the different particles for 24 h were lysed 20 h after infection with *M. smegmatis* and plated on LB agar. Irrespective of species, particle type and concentration we did not observe any mycobacterial growth (Fig. 5, see also Additional file 2) indicating that the cells retained their bactericidal activity.

## Discussion

Magnesium and magnesium-based alloys are currently under intense investigation for the development of biodegradable implants (Moravej and Mantovani 2011; Seitz et al. 2014; Waizy et al. 2013). The degradation process begins when magnesium comes into contact with body fluids. Several kinds of degradation products have been suggested to be biologically relevant, i.e., magnesium ions and alloy metal ions, hydroxide ions, hydrogen gas and abraded particles (Gu et al. 2009). Magnesium ions are essential and highly biocompatible; hydroxide ions are thought to be buffered to maintain a physiological pH while excessive hydrogen evolution can lead to air pockets in the tissue. Particulate implant debris can induce the production of proinflammatory mediators and pathologies like aseptic implant loosening (Hallab and Jacobs 2009; Kaufman et al. 2008). In the present study we investigated whether magnesium-based particles could affect immune functions of macrophages. Macrophages are among the first cells to arrive at the site of injury where they modulate inflammatory and wound healing processes (Koh and DiPietro 2011). They also take up particulate debris which may result in an inflammatory response (Hallab and Jacobs 2009).

Here, the effects of magnesium-based particles on macrophage viability were minor except when excessive amounts of magnesium corrosion particles were added. Previous studies also reported cytotoxic effects of excessive amounts of Mg particles on a rat osteosarcoma cell line (Di Virgilio et al. 2011; Grillo et al. 2011). The reason for this outcome in our study is unclear as it appeared to be independent of magnesium ion concentration and pH. However, it seems conceivable that this effect might be associated with the heterogeneous size distribution of MCP (comprising a number of particles in the low μm range; see Fig. 1) compared to magnesium particles, because particle size has been suggested to be an important determinant of inflammatory potency (Caicedo et al. 2013; Hallab and Jacobs 2009). Co–Cr–Mo particles have been shown to increase the release of proinflammatory cytokines such as TNFα from human monocytes/macrophages (Caicedo et al. 2009, 2010), so we used this particle type as a positive control for our experiments using human macrophages. TNFα can be used as a surrogate marker for an inflammatory response because it is known to play an important role in the orchestration of multiple proinflammatory cytokines (Feldmann and Maini 2003; Parameswaran and Patial 2010). This cytokine was not detectable in the supernatants of murine macrophages treated with magnesium-based particles suggesting that these particles alone are not proinflammatory. Likewise, in human macrophages CCM particles led to an increase in TNFα secretion, whereas magnesium-based particles did not. Considering the moderate release of magnesium ions from the particles this finding is in accordance with a previous study reporting that magnesium ions did not increase TNFα secretion from dendritic cells (Feser et al. 2011).

It is worth noting that CCM particles were smaller and more sharp edged than the magnesium-derived particles used in this study. It has been suggested that debris must be phagocytosable, i.e., less than 10 µm, to induce an inflammatory response, and that irregular surface structures are more inflammatory than smooth ones (Caicedo et al. 2013; Hallab and Jacobs 2009). These features likely explain the inflammatory potential of CCM particles. At least a subset of the Mg corrosion particles also met these physical criteria but yet they did induce a significant secretion of TNFα. One explanation may be that the ongoing corrosion may have mitigated the proinflammatory potential of the magnesium corrosion particles. Moreover, it has been reported that CCM particles activate the inflammasome (Caicedo et al. 2009) thus triggering the release of proinflammatory mediators. MCP may lack this property but this remains to be tested in future investigations. Macrophages are essential effectors of innate immunity and it is, therefore, of utter importance that they maintain their functionality even after the ingestion of implant debris. Apart from their phagocytic activity these cells can process and present antigen to other immune cells, thus triggering the adaptive immune responses (Schenk et al. 2014; Unanue et al. 1969). Here, pretreatment with magnesium particles did neither enhance nor inhibit proliferation of allogenic T cells in a mixed leukocyte reaction, indicating unchanged T cell response. This is in agreement with previous findings where soluble magnesium degradation products did not affect the in vitro immunogenicity of primary dendritic cells (Feser et al. 2011). Notably, the same was true for CCM particles, which corresponds to previous observations that, unlike soluble metal ions, Co–Cr–Mo debris failed to upregulate costimulatory molecules on monocytes (Caicedo et al. 2010).

Among the essential tasks of macrophages is the killing of invading bacteria so any restriction of this ability due to biomaterial implantation must be avoided. Mycobacterial infection of macrophages induces cytokine production and enhances the phagosomal superoxide burst to fight the pathogen (Podinovskaia et al. 2013; Roach and Schorey 2002). After incubation with particles both human and murine macrophages retained bactericidal activity, indicating that this important antibacterial activity was not compromised by magnesium degradation products. Interestingly, TNFα production after infection showed species-specific differences. After incubation with magnesium-derived particles, TNFα was diminished in mouse cells but not in human macrophages. Similar distinctions between murine and human macrophages have been observed previously (Podinovskaia et al. 2013). Human data showed a remarkable degree of variability which might be due to between-donor variation or to unrecognized, subclinical affections of some blood donors. However, there was not even a tendency towards reduced TNFα levels after pretreatment with magnesium-derived particles plus infection. Thus, the differences we observed may rather be linked to the decreased viability of murine cells after particle contact plus infection. If the cells became damaged early after infection, they may have not been able to produce significant amounts of TNFα afterwards. Unfortunately, the limited availability of human blood products did not allow us to test the viability of human macrophages after infection. Apart from the above mentioned, the contrasting results may also be due to differences in the macrophage isolation procedures between mice and men (bone marrow derived vs. blood derived) or to species-specific properties as exemplified by a recent study providing evidence of significant dissimilarities in the genomic response of inflammatory diseases (Seok et al. 2013). Irrespective of the actual reason, the fact that all macrophages remained capable of killing intracellular microorganisms suggests that a diminished TNFα response did not have major detrimental effects on the overall immune function of the cells.

## Conclusions

In the present study immunomodulatory effects of particulate magnesium implant debris on primary macrophages were investigated. The particles were biocompatible and induced neither an exaggerated immune reaction nor did they reveal any immunosuppressive properties. In agreement with in vivo observations, magnesium implant debris did not affect relevant immune functions of macrophages.

## Electronic supplementary material

Below is the link to the electronic supplementary material.

Supplementary material 1 Viability of murine macrophages post infection (PDF 69 kb)

Supplementary material 2 Absence of mycobacterial growth after treatment of macrophages with different particles at different concentrations (PDF 194 kb)
