# Supplementary material for: Magnesium corrosion particles do not interfere with the immune function of primary human and murine macrophages
Source: Prog Biomater. 2014 Dec 6;4:21–30. doi: 10.1007/s40204-014-0032-9 (PMC5151114; doi:10.1007/s40204-014-0032-9)

Additional file 1

Viability of particle-treated murine macrophages after infection

Murine macrophages were incubated for 24 hours with media containing either no particles or indicated amount of magnesium particles (Mg) or magnesium corrosion particles (MCP) before infection with *Mycobacterium smegmatis*. Particle incubation was then continued for further 20 hours before metabolic activity was assessed. Mean ± SD of n = 3. ANOVA, ***p < 0.001 compared to untreated control cells.

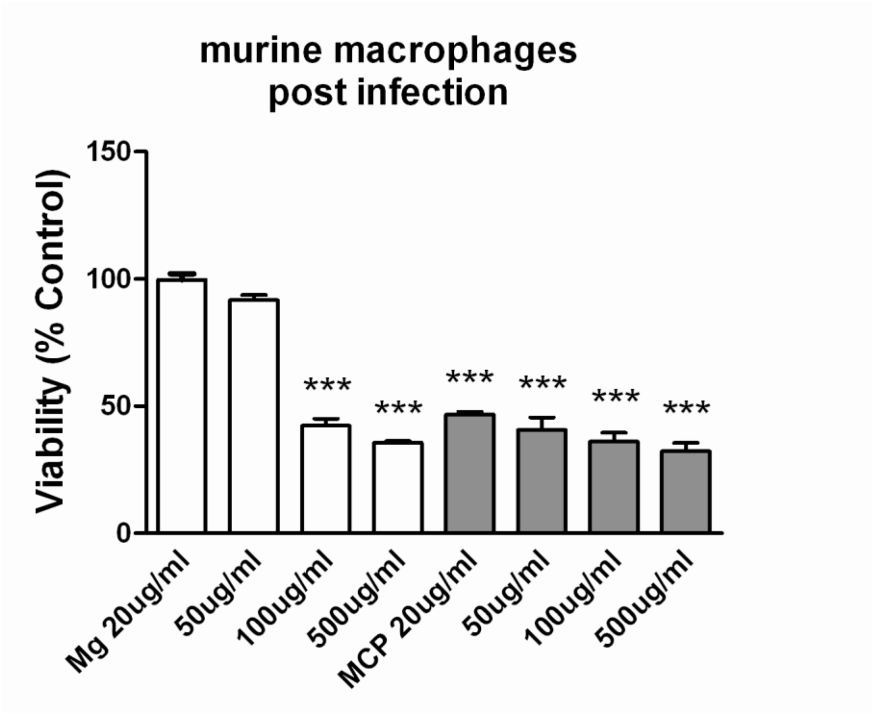

Supplement: Supplementary file 1 — Supplementary material 1 Viability of murine macrophages post infection (PDF 69 kb) [file 40204_2014_32_MOESM1_ESM.pdf]
