# Supplementary material for: Magnesium corrosion particles do not interfere with the immune function of primary human and murine macrophages
Source: Prog Biomater. 2014 Dec 6;4:21–30. doi: 10.1007/s40204-014-0032-9 (PMC5151114; doi:10.1007/s40204-014-0032-9)

Additional file 2

Absence of mycobacterial growth

Human macrophages were incubated for 24 hours with media containing indicated amount of particles before infection with *Mycobacterium smegmatis*. Particle incubation was then continued for further 20 hours before cells were lysed, seeded on Lysogeny broth agar plates and incubated for six days. No colony-forming units were observed in any of the experiments. The plates are representative of at least five independent experiments using human or murine macrophages, respectively.

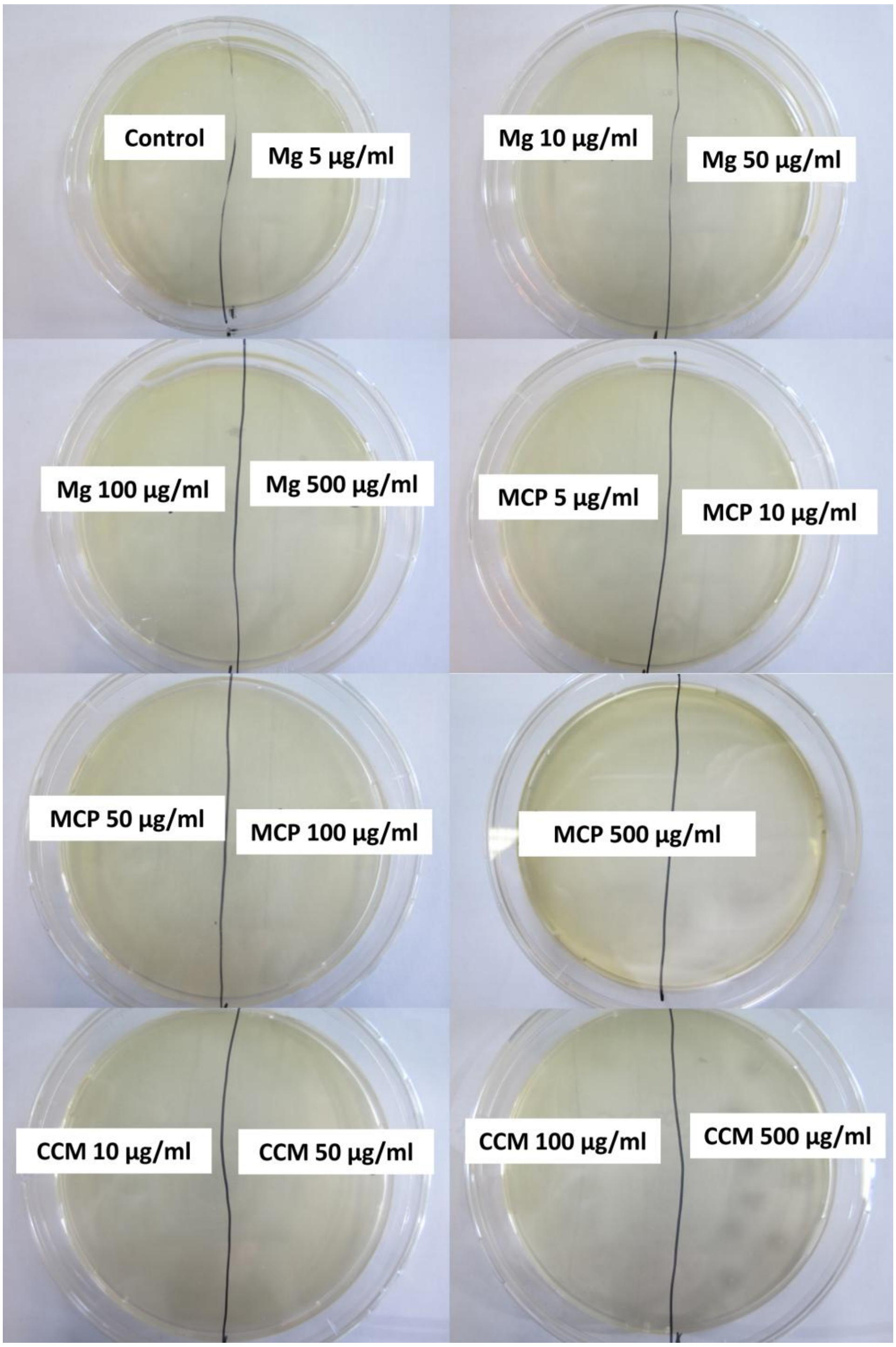

Supplement: Supplementary file 2 — Supplementary material 2 Absence of mycobacterial growth after treatment of macrophages with different particles at different concentrations (PDF 194 kb) [file 40204_2014_32_MOESM2_ESM.pdf]
